# Benign mature teratoma of the cecum: a case report

**DOI:** 10.1093/jscr/rjad674

**Published:** 2023-12-23

**Authors:** Jaclyn Gellings, Kaitlyn Nimmer, Saryn Doucette, Jennifer R Merrill, Salma A Sheriff, Scott C Johnson, Anai N Kothari

**Affiliations:** Division of Surgical Oncology, Department of Surgery, Medical College of Wisconsin, Milwaukee, WI 53226, United States; Division of Surgical Oncology, Department of Surgery, Medical College of Wisconsin, Milwaukee, WI 53226, United States; Department of Pathology, Medical College of Wisconsin, Milwaukee, WI 53226, United States; Division of Surgical Oncology, Department of Surgery, Medical College of Wisconsin, Milwaukee, WI 53226, United States; Division of Surgical Oncology, Department of Surgery, Medical College of Wisconsin, Milwaukee, WI 53226, United States; Department of Urology, Medical College of Wisconsin, Milwaukee, WI 53226, United States; Division of Surgical Oncology, Department of Surgery, Medical College of Wisconsin, Milwaukee, WI 53226, United States

**Keywords:** cystic teratoma, cecum, mature teratoma, pericecal cystic lesion, primary mature teratoma

## Abstract

A teratoma is a typically benign tumor derived from more than one embryonic cell line, and it is characterized by presence of tissue foreign to the tumor location site. With the unlikely primary location in the gastrointestinal tract and no history of malignancy, we present a rare case of a primary mature cystic teratoma of the cecum. The patient is a 66-year-old male with imaging demonstrating an extraluminal, seemingly fat-containing mass abutting the cecum. The patient underwent resection, and final pathology revealed a mature cystic teratoma. Primary mature teratoma of the cecum is exceptionally rare; thus, diagnosis can be challenging. As he had no primary testicular or retroperitoneal mass, this cystic lesion likely represents a developmental abnormality and not a true neoplasm. The radiographic features, presentation, differential diagnoses, and treatment recommendations are discussed.

## Introduction

A teratoma is a typically benign tumor derived from more than one embryonic cell line, and it is characterized by the presence of tissue foreign to the tumor location site [1, 3]. Teratomas may be solid, cystic or mixed, and typical locations include the gonads and mediastinum [1, 3]. Teratomas are extremely rare in males, all the more so when presenting without an associated cancer. Furthermore, the gastrointestinal tract is an exceptionally rare primary location for teratomas, and thus we present a case of a primary mature cystic teratoma of the cecum.

## Case report

**Figure 1:**
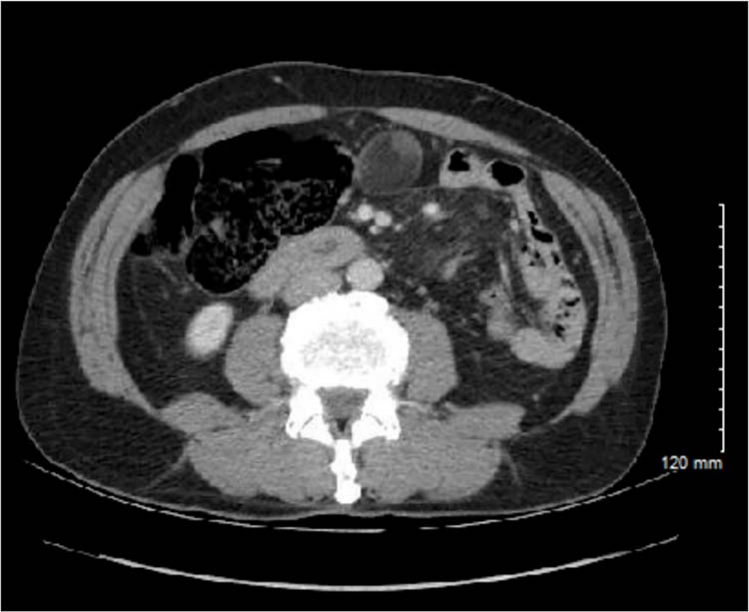
Initial CT imaging showing an extraluminal mass, measuring 3.6 × 3.0 × 3.9 cm with mural soft tissue nodularity within the midline anterior abdomen.

The patient is a 66-year-old male with pertinent medical history including coronary artery disease with recent coronary stent placement who presented to the Emergency Department for evaluation of right flank pain. Cross sectional imaging showed no acute intra-abdominal process; however, an extraluminal, seemingly fat-containing, 3.9 cm mass abutting the cecum and appendix in the midline anterior abdomen was noted (Fig. 1). Differential diagnoses at that time included mesenteric cysts, appendiceal mucocele, nonpancreatic pseudocyst, enteric duplication, epidermoid cyst, and cystic teratoma. Further workup and treatment were unable to be pursued due to elevated risk of bleeding from recent coronary stent placement and antiplatelet treatment with Ticagrelor. Thus, the decision was made for close interval surveillance.

**Figure 2:**
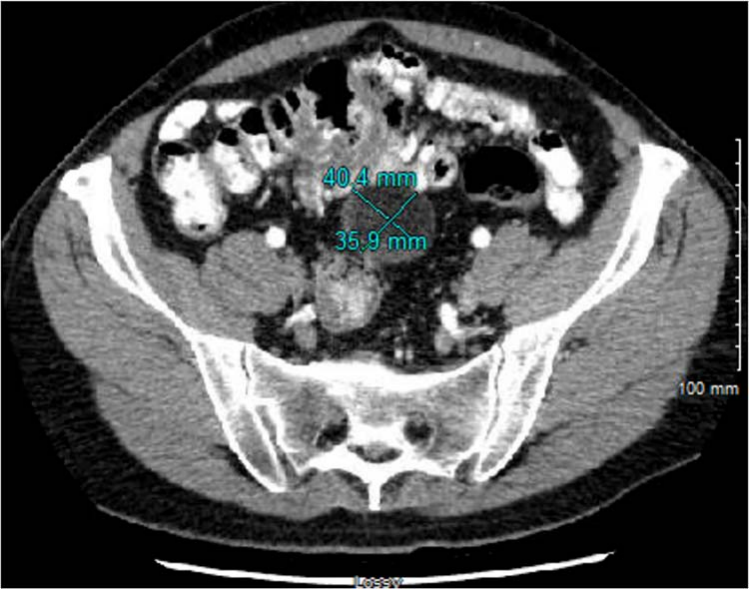
CT imaging from 4 months after initial diagnosis, showing a 4.0 × 3.6 × 3.8 cm, seemingly lipomatous mass in the midpelvis.

The patient underwent serial cross-sectional imaging that demonstrated stable appearance of the mass with migration of the redundant cecum and associated mass into the pelvis (Fig. 2). Colonoscopy showed a normal cecal base and no concerning features apart from sigmoid colon polyps. Due to the location, percutaneous biopsy was considered but ultimately was non-feasible due to anatomic constraints.

**Figure 3:**
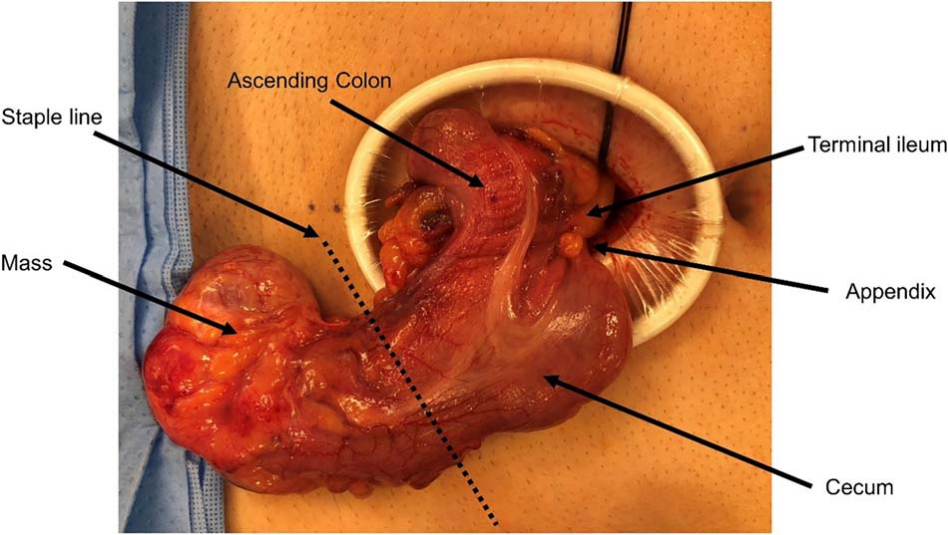
Intraoperative imaging of cecal cystic lesion.

Therefore, the patient underwent planned, hand-assisted laparoscopic resection of the cecal mass and appendectomy. At the time of surgery, the abdomen was accessed laparoscopically, and the mass was revealed in the pelvis after mobilization of the omentum and small bowel. The mass itself was soft and appeared to arise directly from the lateral wall of the cecum as it transitioned into the right colon (Fig. 3). It was clearly distinct from both the terminal ileum and appendix. The mass was eviscerated through an infraumbilical incision, and a partial cecetomy and appendectomy en bloc with the mass was performed.

The patient had an uneventful post-operative course, and he was discharged home on post-operative day 2. After pathologic confirmation of mature cystic teratoma, scrotal ultrasound was found to be unremarkable.

## Pathologic findings

**Figure 4:**
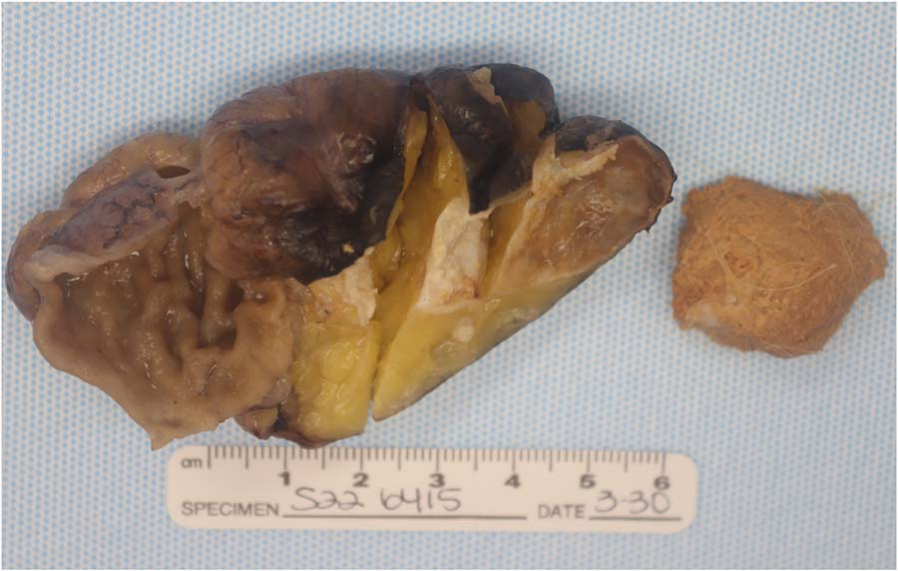
Gross pathology specimen showing a smooth walled cyst to the right of opened colon. The cyst was encased in fat and had no connection to the wall of the colon. There are no solid areas identified in the cyst. Separately, there is a ball of sebaceous material and hairs.

**Figure 5:**
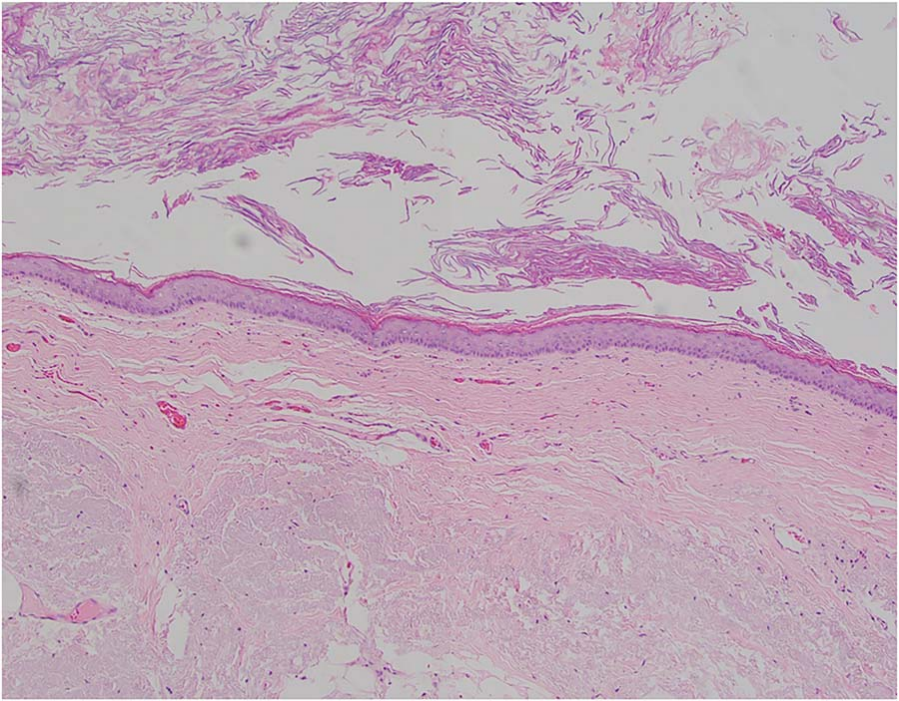
Pictomicrograph of an H&E-stained section of the cyst taken at 10× magnification. No solid areas were identified in the cyst, and no other tissue types were identified besides squamous epithelial lining within a thin wall of loose connective tissue. No cartilage, bone, or glandular tissues were identified. No immature or embryonic-type tissues were identified.

The specimen contained a 4.5 × 3.5 × 2.6 cm soft tissue mass with intact serosal covering encased within the pericolonic adipose tissue (Fig. 4). The specimen had no connection to the muscular wall of the colon, though there was a ball of sebaceous material and hair. Importantly, no solid areas were identified in this cyst, and no other tissue types were identified microscopically besides the squamous epithelial lining within a thin wall of loose connective tissue. Further testing, including histochemical staining and immunohistochemistry, revealed mature cystic teratoma in the pericolic fat with uninvolved margins (Fig. 5).

## Discussion

Teratomas are typically benign tumors which involve multiple germ cell layers; histologic variants include mature teratoma, immature teratoma, teratoma with malignant transformation, and monodermal teratoma [1]. Mature teratoma is a benign variant that is usually cystic [1], as seen in our patient. Typically, the gonads are the most commonly affected location, as explained by the close relationship of the gonads with the migration of germ cells during development [2]. In men, teratomas with mature, somatic-type tissue can metastasize, often to retroperitoneal lymph nodes, followed by visceral and mediastinal metastasis [5]. Pathologic evaluation of the metastatic lesion can show mature elements, immature elements, and malignant transformation [6]. Thus, evaluation of the testes, mediastinum and retroperitoneum is important to exclude possible metastatic teratoma. Notably, our patient did have ultrasound evaluation of his scrotum after pathologic diagnosis of mature teratoma, which was negative for any mass.

In our patient, the progression of cross-sectional images over an ~6-month period showed stable appearance of the mass with gradual migration of the cecum and the mass from the right upper quadrant into the pelvis. At the index computed tomography scan, there was an apparent extraluminal mass abutting the cecum and appendix, and follow-up MRI showed radiographic solid components to the previously described cystic lesion. The solid component was concerning for a fat-containing malignancy within the mesentery of the cecum. Given the uncertainty of the etiology and the patient’s symptoms, surgical resection was recommended. Of note, there was an extensive preoperative cardiac workup, including management of his anti-platelet treatment, and the patient was cleared for surgery.

Due to the rarity of cecal teratomas, diagnosis can be challenging. Clinical presentation may include rectal bleeding, abdominal pain, bowel obstruction, or nontender palpable masses [1–4], and radiologic evaluation may detect calcifications. Tabuchi *et al.* argue that if a colonic tumor shows calcifications on imaging, the diagnosis of primary teratoma should be strongly considered [7]. Differential diagnoses can include mesenteric cysts, lymphatic cyst, appendiceal mucocele, nonpancreatic pseudocyst, enteric duplication, epidermoid cyst, and cystic teratoma [4]. Scheutz *et al.* also characterize the differential diagnoses of pericecal cystic lesions according to their cyst lining and contents, and their description of stratified squamous epithelium lining a mature teratoma is consistent with our patient’s pathologic evaluation.

In our patient, diagnostic work-up was challenging due to the patient’s recent stent placement. Ultimately, the patient presented to the operating room with some diagnostic uncertainty, though his lesion was strongly felt to be primarily located in the cecal mesentery. In this case, without a primary testicular or retroperitoneal mass, this cystic lesion likely represents a developmental abnormality and not a true neoplasm. Within the literature, there exist very few reports of cases of teratoma of the bowel. Treatment in most cases includes local resection, and prognosis is excellent as resection has been curative in all reported cases [1]. As previously discussed, the tumor did arise from the wall of the cecum, though it was entirely extraluminal. The complete excision of the mass is considered curative, and no other follow-up or surveillance is necessary.
